# Dysbiosis Disrupts Gut Immune Homeostasis and Promotes Gastric Diseases

**DOI:** 10.3390/ijms20102432

**Published:** 2019-05-16

**Authors:** Devinder Toor, Mishi Kaushal Wasson, Prashant Kumar, G. Karthikeyan, Naveen Kumar Kaushik, Chhavi Goel, Sandhya Singh, Anil Kumar, Hridayesh Prakash

**Affiliations:** 1 Amity Institute of Virology and Immunology, Amity University, Sector 125, Noida 201313, Uttar Pradesh, India; dtoor@amity.edu (D.T.); mwasson@amity.edu (M.K.W.); pkumar18@amity.edu (P.K.); gkarthikeyan@amity.edu (G.K.); nkkaushik@amity.edu (N.K.K.); cgoel@amity.edu (C.G.); 2 Department of Animal Biology, School of Life Sciences, University of Hyderabad, Hyderabad 500046, Telengana, India; sandhya_singh1@yahoo.com; 3 National Institute of Immunology, Aruna Asaf Ali Marg, New Delhi 110067, India; anilk@nii.ac.in

**Keywords:** gut microbiota, macrophages, TLR mimicry, immune epigenetics, metabolism, sterile inflammation

## Abstract

Perturbation in the microbial population/colony index has harmful consequences on human health. Both biological and social factors influence the composition of the gut microbiota and also promote gastric diseases. Changes in the gut microbiota manifest in disease progression owing to epigenetic modification in the host, which in turn influences differentiation and function of immune cells adversely. Uncontrolled use of antibiotics, chemotherapeutic drugs, and any change in the diet pattern usually contribute to the changes in the colony index of sensitive strains known to release microbial content in the tissue micromilieu. Ligands released from dying microbes induce Toll-like receptor (TLR) mimicry, skew hypoxia, and cause sterile inflammation, which further contributes to the severity of inflammatory, autoimmune, and tumorous diseases. The major aim and scope of this review is both to discuss various modalities/interventions across the globe and to utilize microbiota-based therapeutic approaches for mitigating the disease burden.

## 1. Introduction

An organism is not just an organism but a niche of a large number of communities. The harmony between these communities or biosis determines the health of that organism. From a bacterium, a unicellular system, to the most sophisticated and successful animal of the ecosystem, Homo sapiens are obliged to various other ecological partners on which they depend for a healthy life. The human body harbors a large number of microbiota—on skin, gut, genitals, and other tissues—which are beneficial and are involved in a variety of vital functions. They protect the body from the penetration of pathogenic microbes. These beneficial microbial colonies compete with one another for space and resources. Among various partners, it is human and bacteria which have evolved together during the course of evolution, and symbiosis among them, in the gut, is vital for overall health of an individual. These microbiota contribute to the metabolism and nutrition which are important for human health and, therefore, a balance in the composition of these commensal organisms [1] is crucial to maintain the homeostasis. Intestinal or gut microbes also assist with endogenous turnover of vitamin B complex and other nutrients like minerals and amino acids metabolism and turnover. The exact composition of the human microbiome, which is important for the homeostasis, still remains largely elusive. The best studied gastrointestinal tract (GI tract) microbiota, which comprises viruses, bacteria, fungi, and protozoa, is estimated to be nearly 100 trillion in numbers [2]. Recent studies have provided compelling evidence demarcating good and bad microbiota. Good microbiota interacts with the immune system and keeps their activity at bay contributing to the homeostatic mechanism. However, bad gut microbiota interacts with the immune system differentially and promotes non immunogenic hyper inflammatory reactions which disturb the homeostasis and promote various gastric diseases. Frequent or uncontrolled use of antibiotics, chemotherapeutic drugs, and change in dietary pattern have shown to disrupt the microbiome, leading to disturbance in microbiota or dysbiosis or dysbacteriosis [3] characterized with an imbalance of life-supportive microbes. Among various organs, the gastrointestinal tract, being the most populous, is the most sensitive to being affected by dysbiosis.

A recent meeting of WHO has suggested that degree of dysbiosis can control the severity of various diseases including metabolic, obesity, malnutrition, diabetes, and chronic inflammatory diseases such as inflammatory bowel disease (IBD), and encompassing ulcerative colitis (UC) and Crohn’s disease (CD) [4]. Although various advanced technology platforms like high-throughput sequencing technologies (HTS) as well as genomic techniques have enabled us to understand the influence of the gut microbiome on human health and disease, our knowledge regarding dysbiosis-driven pathogenesis of gastric disease is still in infancy. In view of this, it is important to explore various molecular and immunological aspects of dysbiosis to better understand the clinical relevance of gut microbiome and disease pathogenesis. In this review, we uncovered the state-of-the-art tools that can be exploited to study the gut microbiome with special emphasis on gastrointestinal (GI) diseases, and also put forward therapeutic strategies involving manipulation of gut microbiota by probiotics and various immunoregulatory mechanisms [5] for the management of dysbiosis.

## 2. Eco-Physiological Balance of Gut Microbiota with Gut-Associated Tissues

The gastrointestinal tract in human beings acts as an interface between the host body and antigens/environmental factors. The number of bacterial cells inhabiting the GI tract is almost equal to the total number of cells in the human body, and their genomic content is approximately 10 times more than the human genome [6]. The data generated by Human Microbiome Project and MetaHIT reveals the presence of approximately 2200 different microbial species in the human gut which are divided into 12 bacterial phyla and 1 archaean taxon [7]. Most of these species belong to *Proteobacteria, Firmicutes, Actinobacteria*, and *Bacteroidetes* phylum, and their distribution varies with age of individual [7]. 

**Figure 1:**
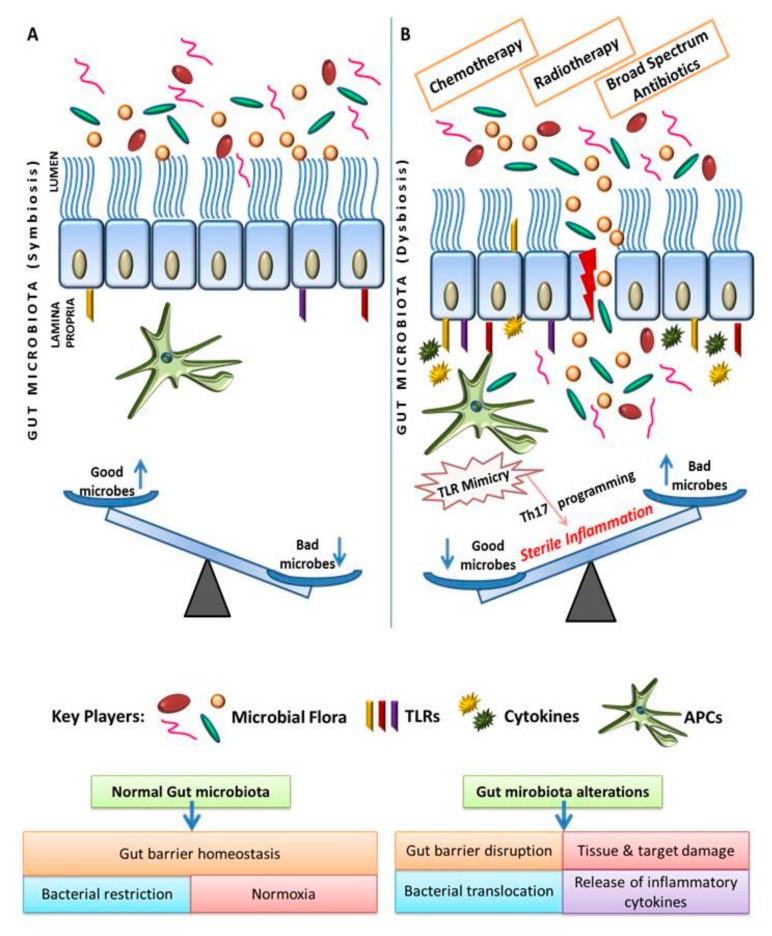
Disruption of normal gut tissue micromilieu including intestinal villi and gut epithelium (**A**) during dysbiosis (**B**) perturb gut immune homeostasis and GI colony index, which is manifested by TLR mimicry, Th17 programming, and hypoxia, which sensitizes normalized gut tissue micromilieu (A) for the progression of gastric inflammatory and tumor diseases.

Normal gut microbiota comprises mostly several genera of Gram-positive Firmicutes and many different Gram-negative bacteroidetes like *Bacteroides, Prevotella, Parabacteroides*, and *Alistipes*. In addition, several other phyla, including the *Proteobacteria, Actinobacteria, Fusobacteria, Verrucomicrobia*, methanogenic archaea, Eukarya (protists and fungi), and other more transient colonizers, are found to be part of the human GI microbiome. Claesson et al. reported that younger individuals between 28 and 46 years of age show higher Firmicutes-to-Bacteroidetes ratio than elderly individuals above 65 years of age [8]. It is further reported that 386 of the identified species are anaerobic and, hence, are located in mucosal regions like the oral cavity and GI tract [7]. Besides exhibiting diversity in composition, the gut microbiota also shows region-/country-specific microbial signatures which further signify that the microbiome is greatly affected by diet as well as the host genetics including age, race, and sex of individuals (Figure 1). However, possibility of functional redundancy between different compositions of gut microbiota cannot be ruled out [9,10]. The host body gets several benefits from these microbes viz. strengthening of gut integrity, food metabolism, protection against virulent pathogens, and regulation of innate immunity [11,12,13,14]. However, if the composition of gut microbiota is altered (dysbiosis), these mechanisms may be disrupted, leading to several inflammatory and other diseases and infections.

The GI tract is colonized by commensal bacteria shortly after birth and the simple bacterial community gradually develops into a complex ecosystem which then starts showing symbiotic relationship with the host [15]. Commensal bacteria, including species of *Bacteroides, Lactobacilli, Bifidobacterium*, and so forth, maintain energy homeostasis by digestion of food components which otherwise cannot be digested by the stomach and intestine of germ-free individuals [16,17]. The gut microbiota also produce a significant amount (~100mM/L per day) of short-chain fatty acids (SCFAs) which act as an energy source for intestinal epithelium and also help regulate gut motility, glucose homeostasis, and inflammation [18,19]. These SCFAs have the capacity to inhibit the growth of enteropathogenic bacteria viz. *S. typhimurium*, enterohaemorrhagic *E. coli*, or *C. rodentium* in the intestine [20]. These commensal microbes also contribute some of the essentially vital vitamins such as cobalamin, vitamin K, riboflavin, biotin, and folates to the host [21].

## 3. Gut Microbiota Plays Critical Role in the Maintenance of Mucosal Immune Homeostasis

The gut microbiota plays an important role in the development of the normal mucosal immune system (humoral and cellular), including the development of gut-associated lymphoid tissues [22,23]. The signaling molecules and metabolites released by commensal microbes are recognized by hematopoietic as well as nonhematopoietic cells of innate immune system which further drive several physiological responses [24]. Reports also indicate that function of gut dendritic cells is largely modulated by tolerogenic response produced by gut microbiota which also inhibits the Th17 anti-inflammatory pathway [25]. Other protective mechanisms of commensal bacteria against invading pathogens include their ability to out-compete pathogens for nutrients and also to produce antimicrobial peptides. Human commensal bacteria like *Bacteroides fragilis* express commensal colonization factors which are required for penetrating the colonic mucus and colonizing the intestinal crypts while another commensal bacterium, *Bacteroides thetaiotaomicron*, expresses corrinoid transporters which help in the uptake of corrinoids that are available in the intestine in limited amount. Competition among these commensal bacteria deprives the invading pathogens of the essential nutrients required for their survival [26,27]. Antimicrobial peptides include bacteriocins like Thuricin CD, which is derived from a commensal bacterium, *Bacillus thurigiensis*, and has the potential to target bacterial pathogens without affecting other commensal bacteria [28]. Commensal bacteria also harbor bacteriophages which confer growth advantage over pathogenic bacteria which do not harbor these bacteriophages [29]. Some other commensal bacteria (e.g., Clostridia species) and the SCFAs produced by them promote proliferation of colonic Treg cells which limit inflammation and maintain intestinal homeostasis [30,31]. However, slight imbalance in the composition of gut microbiota may disturb immune homeostasis, leading to various inflammatory diseases and infections. 

Under normal physiological conditions, symbiotic association of gut microbiota with gut-associated lymphoid tissue (GALT) contributes to immune homeostasis. Both macrophages and M cells of Peyer’s patches play an important role in sensing antigens and transport to mucosal lymphoid nodes for immune responses [32]. Interestingly, gut microbiota colony index contributes to the overall health of the host by promoting the maturation and activation of myeloid cells of GALT, involved in patrolling, to restrict invaders in the gut, thus preventing disease progression. Changes in the composition of microbial communities disrupt gut homeostasis, which promotes leaky gut, inflammatory bowel disease, and allergic inflammation predisposing the affected individuals and making them more sensitive to developing cancer. Intake of broad-spectrum antibiotics efficiently depletes/changes the composition of faecal microbiota and impairs GALT architecture and functions [33]. Gut microbiota also secrete several immunogenic substances such as complex lipopolysaccharides present on the cell surface of Gram-negative bacteria involved in gut immune homeostasis maintenance. *Bacteroides fragilis* represents one class of bacteria found in the human intestine that contributes to immune homeostasis by promoting Foxp3+T cell activity in GALT [34].

Studies in *Bacteroides* have revealed multiple biochemical mechanisms involved in change of gut microbiota index to overcome several challenges posed by the dysbiosis. This may range from variable pH of GI tract to differential oxygen gradients and host immune surveillance. *Bacteroides* depend on other microbes, especially *Ruminococcus obeum*, in the gut to fulfill their need for corrinoid (Vitamin B12 class like cofactors) for their survival, suggesting that corrinoid producers seem to determine the diversity of the gut microbial community, particularly Bacteroides. Germ-free mice, exhibiting immune defects like imbalanced Th1/Th2 response and reduced serum levels of IgA in the gut, are ameliorated by colonization with *Bacteroides* [35], suggesting that host–microbiome interaction has important health implications.

## 4. Change in the Gut Microbiome Triggers Sterile Inflammation and Promotes Gastric Inflammatory Disease

Chronic and recurrent inflammation in the gut triggers oxidative stress which depletes sensitive microbes, leaving resistant strains unaffected. This dysbiosis continuously and adversely agitates GALT to promote sterile inflammatory response and sensitizes the host for chronic gastric disease. Various evidence [36,37,38,39] suggests that changes in intestinal microbiota drive changes in the intercellular tight junctions like desmoglins, facilitate the leaky gut, and enhance the interaction of various danger signals (like) released from the dying bacterial cells with immune cells, thus promoting sterile inflammation. Increasing evidence suggests that dysbiosis is associated with inflammatory bowel disease and a wide range of malignancies. Peyer’s patches (PPs) are surrounded by follicle-associated epithelium (FAE), which forms the interface between the microenvironment of the lumen and the GALT. The FAE consists of specialized M cells that transport antigens and pathogens from the lumen towards underlying immune cells to regulate the immune response. The type of immune response depends upon the interactions between the immune cells located in the FAE and the lymphoid follicle. Immunological tolerance is developed against nonpathogenic normal microflora whereby generation of antigen-specific T cells suppresses activation of the immune system, thus protecting the mucosa from unnecessary inflammation. The gut microbiota and mucosal immunity constantly interact with each other to maintain intestinal homeostasis. However, if this balance is disturbed, dysfunction of the intestinal immune system occurs that further triggers a variety of diseases including IBD. Several studies indicated that intestinal dysbiosis causes an abnormal immune response leading to IBD inflammation and destruction of the gastrointestinal tract. Dysbiosis-driven chronic inflammatory and autoimmune diseases are associated with altered expression of pattern-recognition receptors (e.g., TLRs) and downstream signaling. Both innate immune and non-immune cells, such as intestinal epithelial and stromal cells, sense the pathogen-associated molecular patterns on microbial components mediated by their TLRs. Innate immune cells, such as dendritic cells and macrophages, sense pathogen-associated molecular pattern (PAMP) through TLRs, initiating rapid and effective inflammatory responses against microbial invasion.

Next-generation sequencing technology has enabled us to decipher information about the changes in the microbiome composition of intestinal microflora genome associated with development of the disease. Dysbiosis plays an important role in the development of inflammatory bowel disease (IBD), mainly due to decline in *Firmicutes* and *Bacteroidetes*, and an increase in detrimental bacteria such as *Proteobacteria* and *Actinobacteria* [40]. Due to altered microbial index in IBD, the ability of microbiota to adapt to environmental changes and defend against natural disturbances has been impaired. Therefore, manipulation of intestinal microflora has been a powerful preventive and therapeutic intervention for the management of this disease. These responses may be used as markers for immunomodulation in therapeutic intervention in IBD. A deficiency in IL-10 has been observed with cases of early-onset IBD [41].

Decrease in dietary fibers containing short-chain fatty acids (SCFAs) which are produced by the fermentation of gut bacteria [42] in the fecal samples from IBD patients [43] provides a correlation of dysbiosis with the onset and progression of IBD. The SCFAs are known to regulate inflammatory responses in different ways, such as binding of SCFA receptors (GPR43) and regulation of colonic Treg cell homeostasis [44] by restoring the colonic size and the functioning of the Treg cell pool in germ-free mice. 

Altered composition of *Bacteroides* and *Firmicutes* has been reported in both animal models and in human subjects under disease conditions. Abundance of *Bacteroides* has been found in Estonian and Finnish children suffering from type 1 diabetes (T1D) as compared with Russian children who have lower T1D prevalence [45] but, however, were shown to have abundance of *Bifidobacterium*. This study also demonstrated that lipopolysaccharide (LPS) from *Bacteroides dorei*, the most common *Bacteroides* sp. in Finnish cohorts, promoted immune tolerance and failed to protect nonobesediabetic (NOD) mice from developing T1D as compared with LPS of *E. coli* origin. Metabolism of gut microbiota can also have adverse consequences, as shown by the facilitation of growth of enterohemorrhagic *E. coli* (EHEC), by *Bacteriodes*, especially *B. thetaiotamicron* and *B. vulgatus*, which cleave fucose and sialic acid moieties and other sugars from mucosal glycoproteins that are then consumed by EHEC, leading to enhanced expression of its virulence genes [46].

## 5. Change in the Gut Microbiome Triggers TLR Mimicry and Promotes Cancer-Related Inflammation

The pathogen recognition receptors (PRRs) present on the immune cells recognize the PAMPs on the commensal and pathogenic bacteria and execute immune response. PRRs like the Toll-like receptors (TLRs) and the nucleotide oligomerization domain (NOD) are expressed on follicle-associated epithelial or dendritic cells. 

NOD2 regulates the size, number, and T-cell composition within PPs in response to the gut flora. NOD2 contributes in the immunologic tolerance towards gut microflora and plays an important role in the function of CD4+ T-cells, which in turn are able to modulate the para- and transcellular permeabilities. NOD2 influences the development of the GALT and is also able to modulate the immune response towards bacteria by limiting the development of a Th1 immune response. In wild-type mice DCs, there is proliferation of naïve CD4+ T-cells with a Th2-like cytokine profile, whereas DCs carrying NOD2 mutations promote the development of Th1-orientated cells. In the absence of NOD2, PPs present a higher rate of CD4+ T cells and M cells in the FAE and increase the results into increased levels of Th1 (IFN-γ, TNF-α, and IL-12p70) and Th2 (IL-4) cytokines. These immune alterations are associated with an increase of paracellular permeability and yeast/bacterial translocation [47]. Indeed, PPs from NOD2 −/− mice exhibit an elevated translocation of Escherichia coli, *Staphylococcus aureus*, and *Saccharomyces cerevisiae* [47].

Since gut microbiota lives in close proximity with colonic mucosa, dysbiosis not only influences inflammatory response but also the digestion and other vital functions of the gut. Dysbiosis-driven breakage of tolerance mechanism often leads to chronic inflammation and sensitizes the gut for chronic diseases other than IBD, like cancer, which actually depends upon the desmoplastic reactions mimicked by various microbial products. During dysbiosis, commensal bacteria are remodeled toward pathogenic bacteria which are accompanied by Th17 immune response which promotes pathogenic inflammation as observed in colorectal cancer (CRC).

Pathogenic bacteria like Enterotoxigenic *B. fragilis* promotes inflammation and produces genotoxins, which leads to cell proliferation and mutations, and enhances the colonization of bacterial species like *Fusobacterium* species that promote tumor progression [48]. All these processes are facilitated by the increased permeability of mucosal surfaces which allows bidirectional movement of the gut microbiota and their interaction with immune cells like CD169+/TCR-1+ lumen macrophages, type-2 neutrophiles, and regulatory T cells. This special interaction of these gut microbiota with refractory immune cells promotes desmoplastic reactions which further enhance sterile inflammation and sensitize the host for cancer progression. Most of these events are reported to promote epigenetic changes in neighboring cells.

Activation of PRRs like TLRs (especially TLR2 and TLR4) and NLRs, both by specific and nonspecific mechanisms, leads to TLR mimicry which confers chemo/radio resistance in tumor cells, influenced by the presence of pathogen-derived genotoxic factors like cyclomodulins which favor cellular proliferations and differentiation. During dysbiosis, activation of M1 macrophages contributes to the production of genotoxins in the form of ROS/RNI having toxic properties [48]. The *Fusobacterium* species that promotes the upregulation of noncanonical NF-κB-driven inflammatory genes has also been found in rich amounts in colonic tumors. Most of these mechanisms are similar to the pathogenesis of *H. pylori*-driven gastric cancer, including methylation oflysyl oxidase tumor suppressor gene which promotes tumor generation. Dysbiosis promotes not only bacterial but also virus-associated cancer. The expression of cancer-causing E6 and E7 protein of HPV is affected by estrogen and it has been shown that gut microbiota greatly modulates the blood estrogen level, thus contributing to tumor development [49,50]. Accumulated data suggests that dysbiosis-induced carcinogenesis is multifactorial in nature. Gut microbiota can pervasively dictate cancer progression by inducing desmoplastic reaction which includes sterile inflammation, epigenetic modification of DNA, and subsequent genomic instability of host cells [51].

We [52] and others [53,54,55] have previously demonstrated that many pathogenic microbes which are associated with cancer reprogram macrophages immunologically and metabolically during persistency. Apart from this, many pathogenic bacteria/viruses like *Helicobacter pylori*, *Fusobacterium nucleatum*, and *Chlamydia* sp. in the microbiota potentially alter the cell cycle progression and also inhibit apoptosis [56], thus conferring cancer-like phenotype in persistently infected tissue micromilieu. Moreover, cross-reactivity of gut microbiome or its metabolites with PRRs/TLRs is anticipated to promote TLR mimicry [57] and is believed to influence sterile inflammatory response for promoting cancer progression, as shown in Figure 1. In the same line, *F. nucleatum* can alter chemotherapeutic response in colorectal cancer.

Recent studies have illustrated that several bacteria species, such as members of *Proteobacteria, Firmicutes, Actinobacteria*, and *Fusobacteria* phyla, have been detected in gastric cancer biopsies [58] which may serve as a prognostic factor, thus reinforcing the association of dysbiosis with cancer.

Evidence illustrates that many bacteria like *H. pylori* modulate the host genome, altering different signaling pathways such as TLRs. The TLRs categorized as transmembrane proteins recognize PAMPs which are critical for innate and adaptive immunity. Lipids, nucleic acids, and proteins from dying bacterial, viral, or fungal cells are potent ligands or PAMPS and they interact preferentially with TLR-2,4, 5, and 9 [57] to trigger TLR mimicry and activate distinct signaling pathways. *H. pylori* upregulates TLR4 to facilitate its adherence to gastric epithelial cells and activates NF-κB via TLR5 interaction along with AP-1 and MAP kinases, resulting in expression and secretion of proinflammatory cytokines. Interestingly, hyperactivation of MAPK signaling has been associated with polarization of regulatory macrophages toward cancer-promoting phenotype. Besides altering regulatory pathways, numerous studies proposed that host genetic makeup can influence the interaction of various microbiota with host cells. For instance, genetic variants rs1640827 and rs17163737 of TLR5 lead to enhanced interactions with *H. pylori*, therefore increasing gastric cancer susceptibility [59]. A similar mechanism during dysbiosis is anticipated to lead to a similar response in various diseases. Many bacterial species like *S. bovis*, *Bacteroides fragilis*, *Escherichia coli*, *Enterococcus*, *Shigella*, *Klebsiella, Streptococcus, Peptostreptococcus*, and so forth, which are present in human gut microbiota, have been associated with progression of colorectal cancer (Table 1). Approximately, 25–80% of patients with *S. bovis* bacteremia exhibit CRC-like [60] symptoms and their stool samples have shown a higher population of bacteria belonging to the Bacteroides–Prevotella group.

Intragastric infiltration of Th17 cells like CD169+/TCR-1+ myeloid cells, CD4/fOXp3 Treg, and macrophages and immature DCs skew sterile inflammatory responses which potentially promote neoplastic transformation of infected or inflamed gut (Figure 1) during development of cancer. Thus, it is intriguing to understand the molecular/immunological mechanism and the causal relationship between the immune system and microflora in development of CRC. Studies using cyclophosphamide suggest that selective enrichment of the gut with Gram-positive bacteria can enhance Th1 immune responses and offer therapeutic benefits, indicating that the gut microbiota might promote anticancer immune responses [61].

## 6. Immune Pharmaceutics as Next-Generation Modalities for Breaking Dysbiosis

Diet and geographical location play a major role in determining the microbial diversity in the gut. Uncontrolled use of antibiotics (both prescribed and indiscriminate usage) often kills a broad variety of sensitive gut microbes and leads to dysbiosis which warrants the inclusion of pro- and/or prebiotics to repopulate the gut and modulate the gut microbiome [82]. More than 1200 clinical trials investigating the effect of probiotics either alone or in combination, for various diseases, are listed in the clinical trials database, with several studies completed and in the data analysis stage [83,84]. Probiotics are live microorganisms generally belonging to the genera most commonly found in fermented foods. Probiotics modulate the gut micromilieu mainly by modulating intestinal epithelial signaling pathways, influencing the titer of sIgA and other Th2 effector cytokines, and by enhancing the intestinal epithelial barrier function by virtue of their increased mucin secretion [85]. It is interesting to note that every probiotic follows a particular mechanism for promoting balance or reconstituting health. Certain probiotics like *Lactobacilli* and *Bifidobacterium* have shown to compete with cariogenic species like *Streptococcus mutans*. In a clinical trial, twice daily oral or once weekly intravaginal administration of *Lactobacillus rhamnosus* GR-1 and *Lactobacillus reuteri* has shown to reduce recurrences of UTI and restore a normal lactobacilli-dominated vaginal flora over anaerobes in patients [86]. This is due to their potential to produce more lactose, which is an important nutrient that gets metabolized to glucose and galactose in most neonates. However, intolerance tolactose and its metabolism leads to the alterations in colonic microbiota. In such cases, probiotic supplementation could alleviate lactose intolerance. Such intervention is quite effective in preventing diseases like Eczema, diarrhea, upper RTI, necrotizing enterocholitis, and pulmonary exacerbation in children. Recently, a randomized, double-blind, placebo-controlled trial of *Lactobacillus acidophilus* and Inulin has shown the efficacy in reducing free and LDL bound cholesterol by 7.84 and 9.27%, respectively, in a cohort of obese patients [87]. It is becoming evident that the gut microbiome can actually have a role in obesity and irritable bowel syndrome (IBS) as well. A study reported that if mice are reared in a germ-free environment and have no microorganisms in their gut, such mice are protected from obesity and metabolic disorders like insulin resistance and glucose intolerance even when fed with a western-style diet loaded with high fat or high calories. Along similar lines, another recent study has established the link of specific gut microbiota on the therapeutic efficacy of metformin in the cohort of obese patients over lean cohort [88]. This is what is anticipated to be due to immune metabolic programming of M1 or Th1 macrophages [89] of obese patients towards M2 and/or scavenging macrophages by metformin which is believed to change the specific gut microbiota in obese people, contributing to the outcome.

## 7. Role of Bile Acids and Gut Microbiome

It is becoming increasingly clear that the gut microbiome utilizes the host food/nutrients for synthesizing bioactives that activate the cellular signaling mediated by cognate receptors on human cells. One important class of cholesterol-derived bioactives is the bile acids produced by host liver released into the duodenum after a meal which have been shown to be metabolized further by the resident microbiomes. These bile acid metabolites activate cellular receptors, in the GI tract as well as in the periphery, regulating several host metabolic process. Gut microbiota not only regulates bile acid synthesis, but also its reuptake, thus contributing to the available bile acid pool of the host [71].

Human liver produces cholic acid (CA) and chenodeoxycholic acid (CDCA), while murine liver produces CA and muricholic acid (MCA) which in turn are conjugated to aminoacids [90]. The amphipathic structure of bile acids gives them detergent-like properties that facilitate emulsification and absorption of dietary lipids and fat-soluble vitamins.

Further, it poses a challenge to the resident microbes, and these organisms have evolved a variety of ways to thrive in this environment. Several of these have bile-acid-inducible (BAI) genes. Metagenomics analysis has revealed that bile salt hydrolase (BSH) activity is present in many bacterial species which are the members of *Lactobacilli, Bifidobacteria, Clostridium*, and *Bacteroides* [90]. In reality, BSH activity is enriched in the gut microbiota and is probably responsible for increased resistance to bile toxicity. Another major microbial biotransformation of bile acids is by the hydroxysteroid dehydrogenases (HSDHs) which are present in *Actinobacteria, Proteobacteria, Firmicutes*, and *Bacteroidetes* [91].

### Regulation of Bile Acid Synthesis via FXR: Role of Gut Microbial Metabolites

Synthesis of bile acids is regulated tightly by negative feedback inhibition through the nuclear receptor FXR, expressed at fairly high levels in the liver and ileum. The most potent ligand for FXR is CDCA, followed by CA, DCA, and LCA. Gut microbes have been demonstrated not only to metabolize bile acids but also to effect signaling throughFXR. The microbiota deconjugates the naturally occurring FXR antagonist taurine-conjugated MCA (TMCA) and thus promotes FXR signaling in mice and is also required for the production of secondary bile acids acting as ligands for TGR5 [92]. Bile acids can shape the gut microbiota community by promoting the growth of bile-acid-metabolizing bacteria and by inhibiting the growth of other bile-sensitive bacteria. It suggests that the interaction between the microbiota and bile acids is not unidirectional. Semisynthetic bile acids like obeticholic acid have shown to have clinically meaningful benefit in nonalcoholic fatty liver (NAFLD) patients in clinical trials. In addition, it is now evident that bile acids not only have direct antimicrobial effects, by destroying bacterial membranes due to their detergent properties, but also have indirect effects through FXR, by inducing transcription of antimicrobial agents (e.g., iNOS and IL-18) that affect the gut microbiota via the immune system [91].

## 8. Future Perspective

Although numerous animal and human studies so far have acclaimed the safety and health benefits of probiotics, this area of research is still in its infancy and warrants more rigorous studies to claim its impact on its expected outcome on health. Utilization of microbial-based strategies is expected to afford help in the management of large numbers of haemolytic/metabolic diseases. Industrial application of Microcins, Colicins, plantaricin, vibriocin, and so forth, which are bacteriocins toxins and produced by *E. coli*, *Lactobacillus* sp. have been explored against various bad gut microbiota associated with many diseases. Fecal material transplantation (FMT) represents one such intervention which is explored for the management of various infections and cancer. Transplantation of gut microbiota of “normal” mice into such germ-free mice led to significant weight gain even in the face of a normal diet, suggesting that the microbiome is contributing to this weight gain.

This approach has decisive influence on cancer-directed immune therapies, thus certainly representing a novel biological entity for affording better treatment options. Finally, it would be essential to identify the set of gut microbiota which is responsible in promoting gut immune homeostasis, mainly deciphering their immunomodulatory impact on the gut on the component of innate and adaptive immunity for homeostasis.

## Figures and Tables

**Table 1:** Overview of bacteria which are associated with different types of diseases.

**Microorganism**	Epigenetic Modifications	Disease
*Enterococcus faecalis*	Extracellular superoxide causing DNA breaks	CRC [62]
*Shigella*	Inflammation	CRC [63]
*Escherichia coli*	Syntheses of toxins	CRC [64]
*Bacteroidesfragilis*	Toxin production Inflammatory response by Th17/IL-17	CRC [65]
*Streptococcus bovis*	Inflammation	CRC [66]
*Helicobacter pylori*	Syntheses of toxins, DNA damage, p53 degradation	CRC [67]
*Fusobacterium nucleatum*	Modulates the tumor immune microenvironment	CRC [68]
*Bifidobacterium*	Decreases b-glucuronidase activity	CRC [69]
*Eubacterium rectale*	Butyrate inducer	CRC [70]
*Clostridium septicum*	Secondary bile acids synthesis	CRC [71]
*Faecalibacterium prausnitzii*	Induces butyrate	CRC [72]
*Lactobacillus*	Decreases lactic acid; activation of Toll-like receptors	CRC [73]
*Bacteroides fragilis*	TLR2 ligand, orchestrates anti-inflammatory immune responses, stimulatesFoxp3C Treg cells	Colitis [74]
*Faecalibacterium prausnitzii*	Inhibits NF-kB activation	Crohn’s disease [75]
*B. thetaiotaomicron*	Attenuates proinflammatory cytokine expression	Colitis [76]
*Salmonella enteric*	Flagellin is recognized by TLR5 which activates proinflammatory pathways in response to infections	Decreased susceptibility to IBD [77]
*Escherichia coli*	NOD2 mutation	Crohn’s disease [78]
*Staphylococcus aureus*	Binds TLR2, inhibits proinflammatory cytokines TNF, IL-12, and IL-6	IBD [79,80]
*Eubacterium rectale, Eubacterium hallii, and Roseburia*	Natural HDAC inhibitors epigenetically activate p21, bax or suppress Cox-2	Cancer [81]

## References

[1] Eloe-Fadrosh E.A., Rasko D.A. (2014). The human microbiome: From symbiosis to Pathogenesis. Annu. Rev. Med..

[2] Leblanc J.G., Milani C., Giori G.S., De Sesma F., Van Sinderen D., Ventura M. (2013). Bacteria as vitamin suppliers to their host: A gut microbiota perspective. Curr. Opin. Biotechnol..

[3] Gareau M.G., Sherman P.M., Walker W.A. (2010). Probiotics and the gut microbiota in intestinal health and disease. Nat. Rev. Gastroenterol. Hepatol..

[4] Kamada N., Seo S.U., Chen G.Y., Núñez G. (2013). Role of the gut microbiota in immunity and inflammatory disease. Nat. Rev. Immunol..

[5] Fraser C.M., Ringel Y., Sanders M.E., Sartor R.B., Sherman P.M., Versalovic J., Young V., Finlay B.B. (2012). Perspective Defining a Healthy Human Gut Microbiome: Current Concepts, Future Directions, and Clinical Applications. Cell Host Microbe..

[6] Sender R., Fuchs S., Milo R. (2016). Revised Estimates for the Number of Human and Bacteria Cells in the Body. PLoS Biol..

[7] Hugon P., Dufour J.C., Colson P., Fournier P.E., Sallah K., Raoult D. (2015). A comprehensive repertoire of prokaryotic species identified in human beings. Lancet Infect. Dis..

[8] Stanton C., Cusack S., O’Mahony D., O’Connor K., Henry C., Greene-Diniz R., Claesson M.J., Fitzgerald A.P., Fitzgerald G., de Weerd H. (2010). Composition, variability, and temporal stability of the intestinal microbiota of the elderly. Proc. Natl. Acad. Sci. USA.

[9] Rodriguez J.M., Murphy K., Stanton C., Ross R.P., Kober O.I., Juge N., Avershina E., Rudi K., Narbad A., Jenmalm M.C. (2015). The composition of the gut microbiota throughout life, with an emphasis on early life. Microb. Ecol. Heal. Dis..

[10] Manichanh C., Bork P., Hansen T., Brunak S., Xu X., Zhong H., Prifti E., Chen W., Sunagawa S., Zhang W. (2014). An integrated catalog of reference genes in the human gut microbiome. Nat. Biotechnol..

[11] Natividad J.M.M., Verdu E.F. (2013). Modulation of intestinal barrier by intestinal microbiota: Pathological and therapeutic implications. Pharmacol. Res..

[12] Van Eunen K., den Besten G., Groen A.K., Reijngoud D., Venema K., Bakker B.M. (2013). The role of short-chain fatty acids in the interplay between diet, gut microbiota, and host energy metabolism. J. Lipid Res..

[13] Bäumler A.J., Sperandio V. (2016). Interactions between the microbiota and pathogenic bacteria in the gut. Nature.

[14] Gensollen T., Iyer S.S., Kasper D.L., Blumberg R.S., Medical H. (2016). How colonization by microbiota in early life shapes the immune system. Science.

[15] Kaetzel C.S., Frantz A.L., Stromberg A.J., Rogier E.W., Bruno M.E.C., Cohen D.A., Wedlund L. (2014). Secretory antibodies in breast milk promote long-term intestinal homeostasis by regulating the gut microbiota and host gene expression. Proc. Natl. Acad. Sci. USA.

[16] Larsbrink J., Rogers T.E., Hemsworth G.R., Mckee L.S., Tauzin A.S., Spadiut O., Klinter S., Pudlo N.A., Urs K., Koropatkin N.M. (2015). Inhibitors of Apoptosis Protein Antagonists (Smac Mimetic Compounds) Control Polarization of Macrophages during Microbial Challenge and Sterile Inflammatory Responses. Nature.

[17] Goh Y.J., Klaenhammer T.R. (2014). Genetic Mechanisms of Prebiotic Oligosaccharide Metabolism in Probiotic Microbes. Annu. Rev. Food Sci. Technol..

[18] Cani P.D. (2013). Gutmicrobiota and obesity: Lessons from the microbiome. Brief. Funct. Genomics.

[19] Valdes A.M., Walter J., Segal E., Spector T.D. (2018). Role of the gut microbiota in nutrition and health. BMJ.

[20] Bohez L., Boyen F., Timbermont L., Ducatelle R., Gantois I., Pasmans F., Haesebrouck F., van Immerseel F. (2006). Oral immunisation of laying hens with the live vaccine strains of TAD Salmonella vac® E and TAD Salmonella vac® T reduces internal egg contamination with Salmonella Enteritidis. Vaccine.

[21] Koutmos M., Kabil O., Smith J.L., Banerjee R. (2010). Structural basis for substrate activation and regulation by cystathionine beta-synthase (CBS) domains in cystathionine –synthase. Proc. Natl. Acad. Sci. USA.

[22] Cebra J.J. (1999). Influences of microbiota on intestinal immune system development. Am. J. Clin. Nutr..

[23] Round J.L., Mazmanian S.K. (2014). The gut microbiota shapes intestinal immune responses during health and disease. Nat. Rev. Immunol..

[24] Malitsky S., Rothschild D., Moresi C., Kuperman Y., Elinav E., Rozin S., Harmelin A., Thaiss C.A., Halpern Z., Levy M. (2016). Persistent microbiome alterations modulate the rate of post-dieting weight regain. Nature.

[25] Magrone T., Perez de Heredia F., Jirillo E., Morabito G., Marcos A., Serafini M. (2013). Functional foods and nutraceuticals as therapeutic tools for the treatment of diet-related diseases. Can. J. Physiol. Pharmacol..

[26] Lee S.M., Donaldson G.P., Mikulski Z., Boyajian S., Ley K., Mazmanian S.K., Engineering B., Jolla L. (2014). Bacterial colonization factors control specificity and stability of the gut microbiota. Nature.

[27] Marrow B., Secreted S., Protect C. (2014). Vitamin B12 as a modulator of gut microbial ecology. Cell Metab..

[28] Vederas J.C., Ross R.P., Rea M.C., Hill C., Sit C.S., Zheng J., Clayton E., O’Connor P.M., Whittal R.M. (2010). Thuricin CD, a posttranslationally modified bacteriocin with a narrow spectrum of activity against Clostridium difficile. Proc. Natl. Acad. Sci. USA.

[29] Rollins D., Clements C.V., Rodrigues J.L.M., Duerkop B.A., Hooper L.V. (2012). A composite bacteriophage alters colonization by an intestinal commensal bacterium. Proc. Natl. Acad. Sci. USA.

[30] Atarashi K., Tanoue T., Oshima K., Suda W., Nagano Y., Nishikawa H., Fukuda S., Saito T., Narushima S., Hase K. (2013). Treg induction by a rationally selected mixture of Clostridia strains from the human microbiota. Nature.

[31] Arpaia N., Campbell C., Fan X., Dikiy S., Liu H., Cross J.R., Pfeffer K., Coffer P.J., Rudensky A.Y., Donald B. (2014). Metabolites produced by commensal bacteria promote peripheral regulatory T-cell generation. Nature.

[32] Da Silva C., Wagner C., Bonnardel J., Gorvel J., Lelouard H. (2017). The Peyer’ s Patch Mononuclear Phagocyte System at Steady State and during Infection. Front. Immunol..

[33] Chemouny J.M., Gleeson P.J., Abbad L., Lauriero G., Bredel M., Bex-coudrat J., Boedec E., Le Roux K., Daugas E., Vrtovsnik F. (2018). Modulation of the microbiota by oral antibiotics treats immunoglobulin A nephropathy in humanized mice. Nephrol. Dial. Transplant..

[34] Telesford K.M., Yan W., Ochoa-reparaz J., Pant A., Kircher C., Christy M.A., Begum-haque S., Kasper D.L., Kasper L.H., Telesford K.M. (2015). A commensal symbiotic factor derived from Bacteroidesfragilis promotes human CD39 C Foxp3 C T cells and T reg function. Gut Microbes.

[35] Kozakova H., Schwarzer M., Tuckova L., Srutkova D., Czarnowska E., Rosiak I., Hudcovic T., Schabussova I., Hermanova P., Zakostelska Z. (2015). Colonization of germ-free mice with a mixture of three lactobacillus strains enhances the integrity of gut mucosa and ameliorates allergic sensitization. Cell. Mol. Immunol..

[36] Conlon M.A., Bird A.R. (2015). The Impact of Diet and Lifestyle on Gut Microbiota and Human Health. Nutrients.

[37] Arrazuria R., Pérez V., Molina E., Juste R.A., Khafipour E. (2018). Diet induced changes in the microbiota and cell composition of rabbit gut associated lymphoid tissue (GALT). Sci. Rep..

[38] Thanabalasuriar A., Koutsouris A., Hecht G., Gruenheid S. (2010). The bacterial virulence factor NleA’s involvement in intestinal tight junction disruption during enteropathogenic E. coli infection is independent of its putative PDZ binding domain. Gut Microbes.

[39] Capaldo C.T., Powell D.N., Kalman D. (2017). Layered defense: How mucus and tight junctions seal the intestinal barrier. J. Mol. Med..

[40] Mohan M., Chow C.T., Ryan C.N., Chan L.S., Dufour J., Aye P.P., Blanchard J., Moehs C.P., Sestak K. (2016). Dietary Gluten-Induced Gut Dysbiosis Is Accompanied by Selective Upregulation of microRNAs with Intestinal Tight Junction and Bacteria-Binding Motifs in Rhesus Macaque Model of Celiac Disease. Nutrients.

[41] Hamilton X.M.K., Boudry G., Lemay D.G., Raybould H.E. (2018). Changes in intestinal barrier function and gut microbiota in high-fat diet-fed rats are dynamic and region dependent. Am. J. Physiol. Gastrointest. Liver. Physiol..

[42] Inohara N., Nun G. (2017). Mechanisms of inflammation-driven bacterial dysbiosis in the gut. Mucosal. Immunol..

[43] Salzer E., Kansu A., Sic H., Dogu F.E., Prengemann N.K., Santos-valente E., Pickl W.F., Demir A.M., Ensari A., Colinge J. (2014). Early-onset inflammatory bowel disease and common variable immunodeficiency—like disease caused by IL-21 deficiency. J. Allergy Clin. Immunol..

[44] Desai M.S., Seekatz A.M., Koropatkin N.M., Stappenbeck T.S., Martens E.C. (2016). A Dietary Fiber-Deprived Gut Microbiota Degrades the Colonic Mucus Barrier and Enhances Pathogen Article A Dietary Fiber-Deprived Gut Microbiota Degrades the Colonic Mucus Barrier and Enhances Pathogen Susceptibility. Cell.

[45] Vatanen T., Kostic A.D., Hennezel E., Cullen T.W., Knip M., Xavier R.J., Huttenhower C., Gevers D., Cullen T.W., Szabo S.J. (2016). Variation in Microbiome LPS Immunogenicity Contributes to Autoimmunity in Humans Article Variation in Microbiome LPS Immunogenicity Contributes to Autoimmunity in Humans. Cell.

[46] Wexler A.G., Goodman A.L. (2017). An insider’s perspective: Bacteroides as a window into the microbiome. Nat. Microbiol..

[47] Barreau F., Meinzer U., Chareyre F., Berrebi D., Niwa-Kawakita M., Dussaillant M., Foligne B., Ollendorff V., Heyman M., Bonacorsi S. (2007). CARD15/NOD2 Is Required for Peyer’s Patches Homeostasis in Mice. PLoS ONE.

[48] Sheflin A.M., Whitney A.K., Weir T.L. (2014). Cancer-Promoting Effects of Microbial Dysbiosis. Curr. Oncol. Rep..

[49] Spurgeon M.E., Den Boon J.A., Horswill M., Barthakur S., Forouzan O., Rader J.S. (2017). Human papillomavirus oncogenes reprogram the cervical cancer microenvironment independently of and synergistically with estrogen. Proc. Natl. Acad. Sci. USA.

[50] Chen K.L., Madak-erdogan Z. (2016). Estrogen and Microbiota Crosstalk: Should We Pay Attention?. Trends Endocrinol. Metab..

[51] Bhat M.I., Kapila R. (2017). Dietary metabolites derived from gut microbiota: Critical modulators of epigenetic changes in mammals. Nutr. Rev..

[52] Nadella V., Mohanty A., Sharma L., Yellaboina S., Mollenkopf H.-J., Mazumdar V.B., Palaparthi R., Mylavarapu M.B., Maurya R., Kurukuti S. (2018). Inhibitors of Apoptosis Protein Antagonists (Smac Mimetic Compounds) Control Polarization of Macrophages during Microbial Challenge and Sterile Inflammatory Responses. Front. Immunol..

[53] Karpiński T. (2019). Role of Oral Microbiota in Cancer Development. Microorganisms.

[54] Xiong Y.B., Zhu H.R., Cheng Y.L., Yu Z.H., Chai N. (2014). Gut microbiome and risk for colorectal cancer. World Chin. J. Dig..

[55] Nagy K.N., Sonkodi I., Szöke I., Nagy E., Newman H.N. (1998). The microflora associated with human oral carcinomas. Oral Oncol..

[56] Gagnaire A., Nadel B., Raoult D., Neefjes J., Gorvel J. (2017). Collateral damage: insights into bacterial mechanisms that predispose host cells to cancer. Nat. Rev. Microbiol..

[57] Frosali S., Pagliari D., Gambassi G., Landolfi R., Pandolfi F., Cianci R. (2015). How the Intricate Interaction among Toll-Like Receptors, Microbiota, and Intestinal Immunity Can Influence Gastrointestinal Pathology. J. Immunol. Res..

[58] Coker O.O., Dai Z., Nie Y., Zhao G., Cao L., Nakatsu G., Wu W.K.K., Wong S.H., Chen Z., Sung J.J.Y. (2018). Mucosal microbiome dysbiosis in gastric carcinogenesis. Gut.

[59] Xu T., Fu D., Ren Y., Dai Y., Lin J., Tang L., Ji J. (2017). Genetic variations of TLR5 gene interacted with Helicobacter pylori infection among carcinogenesis of gastric cancer. Oncotarget.

[60] Zou S., Fang L., Lee M. (2018). Dysbiosis of gut microbiota in promoting the development of colorectal cancer. Gastroenterol. Rep..

[61] Viaud S., Saccheri F., Mignot G., Yamazaki T., Hannani D., Enot D.P., Pfirschke C., Engblom C., Pittet J., Schlitzer A. (2013). The intestinal microbiota modulates the anticancer immune effects of cyclophosphamide. Science.

[62] Boonanantanasarn K., Gill A.L., Yap Y., Jayaprakash V., Sullivan M.A., Gill S.R. (2012). Enterococcus faecalis Enhances Cell Proliferation through Hydrogen Peroxide-Mediated Epidermal Growth Factor Receptor Activation. Infect. Immun..

[63] Gao Z., Guo B., Gao R., Zhu Q., Qin H. (2015). Microbiota disbiosis is associated with colorectal cancer. Front. Microbiol..

[64] Nougayrède J.P., Homburg S., Taieb F., Boury M., Brzuszkiewicz E., Gottschalk G., Buchrieser C., Hacker J., Dobrindt U., Oswald E. (2006). Escherichia *coli* induces DNA double-strand breaks in eukaryotic cells. Science.

[65] Boleij A., Hechenbleikner E.M., Goodwin A.C., Badani R., Stein E.M., Lazarev M.G., Ellis B., Carroll K.C., Albesiano E., Wick E.C. (2015). The bacteroidesfragilis toxin gene is prevalent in the colon mucosa of colorectal cancer patients. Clin. Infect. Dis..

[66] Biarc J., Nguyen I.S., Pini A., Gossé F., Richert S., Thiersé D., Van Dorsselaer A., Leize-Wagner E., Raul F., Klein J.P. (2004). Carcinogenic properties of proteins with pro-inflammatory activity from Streptococcusinfantarius (formerly *S. bovis*). Carcinogenesis.

[67] Blot W.J. (2013). Helicobacter pylori protein-specific antibodies and risk of colorectal cancer. Ann. Intern. Med..

[68] Kostic A.D., Chun E., Robertson L., Glickman J.N., Gallini C.A., Michaud M., Clancy T.E., Chung D.C., Lochhead P., Hold G.L. (2013). Fusobacterium nucleatum Potentiates Intestinal Tumorigenesis and Modulates the Tumor-Immune Microenvironment. Cell. Host. Microbe..

[69] Kim Y., Lee D., Kim D., Cho J., Yang J., Chung M., Kim K., Ha N. (2008). Inhibition of proliferation in colon cancer cell lines and harmful enzyme activity of colon bacteria by Bifidobacterium adolescentis SPM0212. Arch. Pharm. Res..

[70] Balamurugan R., Rajendiran E., George S., Samuel G.V., Ramakrishna B.S. (2008). Real-time polymerase chain reaction quantification of specific butyrate-producing bacteria, Desulfovibrio and Enterococcus faecalis in the feces of patients with colorectal cancer. J. Gastroenterol. Hepatol..

[71] Ridlon J.M., Kang D.J., Hylemon P.B., Bajaj J.S. (2014). Bile acids and the gut microbiome. Curr. Opin. Gastroenterol..

[72] Lopez-Siles M., Khan T.M., Duncan S.H., Harmsen H.J.M., Garcia-Gil L.J., Flint H.J. (2012). Cultured representatives of two major phylogroups of human colonic Faecalibacteriumprausnitzii can utilize pectin, uronic acids, and host-derived substrates for growth. Appl. Environ. Microbiol..

[73] Li Y., Zhang X., Wang L., Zhou Y., Hassan J.S., Li M. (2015). Distribution and gene mutation of enteric flora carrying β-glucuronidase among patients with colorectal cancer. Int. J. Clin. Exp. Med..

[74] Balzola F., Bernstein C., Ho G.T., Lees C. (2010). Inducible Foxp3+regulatory T-cell development by a commensal bacterium of the intestinal microbiota: Commentary. Inflamm. Bowel Dis. Monit..

[75] Sokol H., Pigneur B., Watterlot L., Lakhdari O., Bermúdez-Humarán L.G., Gratadoux J.-J., Blugeon S., Bridonneau C., Furet J.-P., Corthier G. (2008). Faecalibacteriumprausnitzii is an anti-inflammatory commensal bacterium identified by gut microbiota analysis of Crohn disease patients. Proc. Natl. Acad. Sci. USA.

[76] Delday M., Mulder I., Logan E.T., Grant G. (2019). Bacteroides thetaiotaomicron ameliorates colon inflammation in preclinical models of Crohn’s disease. Inflamm. Bowel Dis..

[77] Schultz B.M., Paduro C.A., Salazar G.A., Salazar-Echegarai F.J., Sebastián V.P., Riedel C.A., Kalergis A.M., Alvarez-Lobos M., Bueno S.M. (2017). A potential role of Salmonella infection in the onset of inflammatory bowel diseases. Front. Immunol..

[78] Boshoff A.C., Comprehensive H., Boshoff C. (2011). Adherent-invasive E coli in Crohn disease: Bacterial “agent provocateur”. J. Clin. Invest..

[79] Steinert A., Linas I., Kaya B., Ibrahim M., Schlitzer A., Hruz P., Radulovic K., Terracciano L., Macpherson A.J., Niess J.H. (2017). The Stimulation of Macrophages with TLR Ligands Supports Increased IL-19 Expression in Inflammatory Bowel Disease Patients and in Colitis Models. J. Immunol..

[80] Islander U., Andersson A., Lindberg E., Adlerberth I., Wold A.E., Rudin A. (2010). Superantigenic Staphylococcus aureus stimulates production of interleukin-17 from memory but not naive T cells. Infect. Immun..

[81] Sook Lee E., Ji Song E., Do Nam Y. (2017). Dysbiosis of Gut Microbiome and Its Impact on Epigenetic Regulation. J. Clin. Epigenetics.

[82] Neuman H., Forsythe P., Uzan A., Avni O., Koren O., Medicine F., Szold H., St H., Israel S., College Z.A. (2018). Antibiotics in early life: Dysbiosis and the damage done. FEMS Microbiol. Rev..

[83] Kristensen N.B., Bryrup T., Allin K.H., Nielsen T., Hansen T.H., Pedersen O. (2016). Alterations in fecal microbiota composition by probiotic supplementation in healthy adults: A systematic review of randomized controlled trials. Genome Med..

[84] Sáez-lara M.J., Robles-sanchez C., Ruiz-ojeda F.J., Plaza-diaz J., Gil A. (2016). Effects of Probiotics and Synbiotics on Obesity, Insulin Resistance Syndrome, Type 2 Diabetes and Non-Alcoholic Fatty Liver Disease: A Review of Human Clinical Trials. Int. J. Mol. Sci..

[85] Villena J., Kitazawa H. (2014). Modulation of intestinal TLR4-inflammatory signaling pathways by probiotic microorganisms: Lessons learned from Lactobacillus jensenii TL2937. Front Immunol..

[86] Falagas M.E., Betsi G.I., Tokas T., Athanasiou S. (2006). Probiotics for Prevention of Recurrent Urinary Tract Infections in Women A Review of the Evidence from Microbiological and Clinical Studies. Drugs.

[87] Ooi L., Ahmad R., Yuen K., Liong M. (2010). Lactobacillus acidophilus CHO-220 and inulin reduced plasma total cholesterol and low-density lipoprotein cholesterol via alteration of lipid transporters. J. Dairy Sci..

[88] Brunkwall L., Orho-melander M. (2017). The gut microbiome as a target for prevention and treatment of hyperglycaemia in type 2 diabetes: From current human evidence to future possibilities. Diabetologia.

[89] Lumeng C.N., Bodzin J.L., Saltiel A.R. (2007). Obesity induces a phenotypic switch in adipose tissue macrophage polarization. J. Clin. Invest..

[90] Chiang J.Y.L. (2013). Bile Acid Metabolism and Signaling. Comprehensive Physiology.

[91] Wahlström A., Sayin S.I., Marschall H.U., Bäckhed F. (2016). Intestinal Crosstalk between Bile Acids and Microbiota and Its Impact on Host Metabolism. Cell Metab..

[92] Jia W., Xie G., Jia W. (2018). Bile acid-microbiota cross-talk in gastrointestinal inflammation and carcinogenesis. Nat. Rev. Gastroenterol. Hepatol..

